# Lactate dehydrogenase A negatively regulated by miRNAs promotes aerobic glycolysis and is increased in colorectal cancer

**DOI:** 10.18632/oncotarget.3318

**Published:** 2015-04-10

**Authors:** Jian Wang, Hui Wang, Aifen Liu, Changge Fang, Jianguo Hao, Zhenghui Wang

**Affiliations:** ^1^ Intensive Care Unit, Tianjin Hospital, Tianjin, China; ^2^ Department of General Surgery, Tianjin Public Security Hospital, Tianjin, China; ^3^ Department of Anesthesiology, The Second Hospital Affiliated to Tianjin Medical University, Tianjin, China; ^4^ Advanced Personalized Diagnostics LLC, Alexandria, VA, USA; ^5^ Department of General Surgery, Taiyuan Central Hospital, Shanxi, China; ^6^ Department of Anatomy, Histology and Embryology, Logistics University of CAPF, Tianjin, China

**Keywords:** lactate dehydrogenase A, miRNA, 3′untranslated region, warburg effect, colorectal cancer

## Abstract

Reprogramming metabolism of tumor cells is a hallmark of cancer. Lactate dehydrogenase A (LDHA) is frequently overexpressed in tumor cells. Previous studies has shown higher levels of LDHA is related with colorectal cancer (CRC), but its role in tumor maintenance and underlying molecular mechanisms has not been established. Here, we investigated miRNAs-induced changes in LDHA expression. We reported that colorectal cancer express higher levels of LDHA compared with adjacent normal tissue. Knockdown of LDHA resulted in decreased lactate and ATP production, and glucose uptake. Colorectal cancer cells with knockdown of LDHA had much slower growth rate than control cells. Furthermore, we found that miR-34a, miR-34c, miR-369-3p, miR-374a, and miR-4524a/b target LDHA and regulate glycolysis in cancer cells. There is a negative correlation between these miRNAs and LDHA expression in colorectal cancer tissues. More importantly, we identified a genetic loci newly associated with increased colorectal cancer progression, rs18407893 at 11p15.4 (in 3′-UTR of LDHA), which maps to the seed sequence recognized by miR-374a. Cancer cells overexpressed miR-374a has decreased levels of LDHA compared with miR-374a-MUT (rs18407893 at 11p15.4). Taken together, these novel findings provide more therapeutic approaches to the Warburg effect and therapeutic targets of cancer energy metabolism.

## INTRODUCTION

About 1, 660, 290 new cancer cases and 580, 350 cancer deaths occurred in the United States in 2013 [1]. Colorectal cancer is the second in females and the third most commonly diagnosed cancer in males, with over 1.2 million new cancer cases and 608, 700 deaths have occurred in 2008 [2]. Although death rates of colorectal cancer have declined 3%, over 50, 000 patients die each year in the United States [1, 3]. The overall survival of metastatic CRC is less than 2 years, and current therapeutics for advanced CRC, such as chemotherapy and radiotherapy, have limited efficacy and can barely improve patient survival [3]. Identify new therapeutic targets is imperative for the improvement of therapeutic approaches for patients with CRC.

Most cancer cells predominantly produce energy by a high rate of glycolysis and give rise to enhanced lactate production, instead of by a comparatively low rate of glycolysis followed by oxidation of pyruvate in mitochondria [4, 5]. This preferential use of aerobic glycolysis, which is believed to initially arise as a result of a hypoxic tumor microenvironment and termed the Warburg effect, has attained a core hallmark of many cancers [4]. In cancer cells, enhanced glucose uptake and glycolysis lead to high levels of pyruvate. Cancer cells still derive a significant fraction of their ATP through oxidative phosphorylation. However, the microenvironment of cancer cells always be in hypoxic conditions, thus oxidative phosphorylation-dependent production of ATP appears secondary to the use of mitochondrial enzymes. Cancer cells reprogram their metabolism, and this metabolic reprogramming in cancer cells is regulated by several oncogenic genes, including the PI3K/Akt, Myc, or hypoxia-inducible factor (HIF) that serve to increase glucose uptake, glycolysis, and transcription of LDHA [6–10].

The lactate dehydrogenase A (LDHA) is an enzyme which is encoded by the LDHA gene, located on the short p arm of chromosome 11 (11p15.4), plays a critical branch point in metabolism of tumor cells [11–15]. LDHA catalyzes the inter-conversion of pyruvate and L-lactate, accompanying with NADH and NAD^+^ conversion. LDHA has been shown correlation with clinicopathologic features and survival of patients with pancreatic cancer, renal cell carcinoma, esophageal squamous, and gastric cancer [12, 16–21]. Transcript expression and post-transcriptional modification of LDHA is regulated by several known oncogenes and deacetylases, such as Myc, HIF-1α, forkhead box protein M1 (FOXM1), Krüppel-like factor 4 (KLF4), and Sirt2 [9, 12, 14, 16, 22, 23]. The c-Myc transcription factor directly binds the promoter of LDHA in an E-box-dependent manner and virtually activates its transcript expression [23]. Interplay between N-Myc and HIF-1α can significantly activate the transcription of multiple glycolytic genes including LDHA [22]. In embryonal carcinoma P19 cells, HIF-1α interacts with the enhancer and promoter of LDHA, and mutation of a HIF-1α binding site abolished the hypoxic inducibility [9]. In pancreatic cancer cells, FOXM1 binds directly to the LDHA promoter region and activates the expression of LDHA, led to increased cell growth and metastasis [16]. However, there is a negative correlation between KLF4 and LDHA expression in pancreatic cancer cells, and KLF4 binds directly to the promoter regions of the LDHA gene and negatively regulates its transcription activity [12]. Furthermore, deacetylated at lysine 5 (K5) of LDHA by Sirt2 activates LDHA activity, and increases pancreatic cancer cell proliferation and migration [14, 24]. All these findings indicated that LDHA could be a promising therapeutic target.

Recently, several studies has shown that miRNAs may play important roles in cancer metabolism, including glycolysis [25, 26]. However, the exhaustive numbers and underlying mechanisms of miRNAs in glycolysis are unknown. Here, we sought to determine the roles of LDHA expression in aerobic glycolysis in colorectal cancer cells, and identify miRNAs associated with LDHA.

## RESULTS

### High expression of LDHA in human CRC tissues

**Figure 1:**
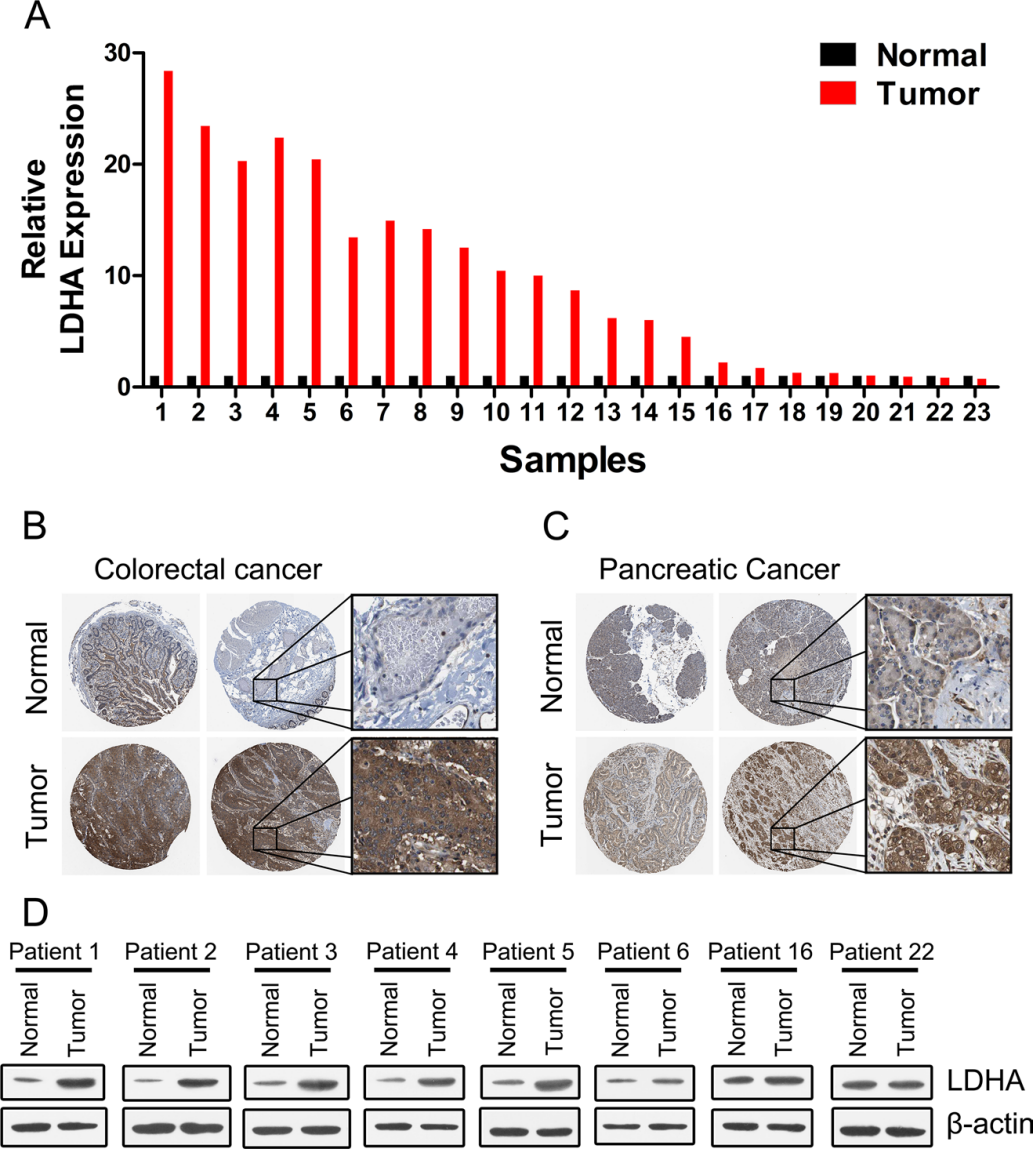
Expression of LDHA in human colorectal cancer and pancreatic cancer tissues **(A)** RT-PCR analysis of LDHA in human colorectal cancer and adjacent normal tissues (*n* = 30). **(B, C)** Higher expression of LDHA was confirmed by immunohistochemistry in human colorectal cancer (B) and pancreatic cancer (C). **(D)** Western blot analysis of LDHA in eight selected human CRC and adjacent normal tissues.

We first examined the expression of LDHA in 30 pairs of human colorectal cancer tissues and matched adjacent normal tissues by RT-PCR. The majority (19/23, or 82.6%) of colorectal cancer tissues (tumor) exhibited higher expression level of LDHA compared to their matched adjacent normal tissues (normal) (Figure 1A). Higher expression levels of LDHA protein in colorectal cancer tissues were also confirmed by immunohistochemistry (Figure 1B). Numerous studies had shown that LDHA is overexpressed in pancreatic cancer and regulates aerobic glycolysis of pancreatic cancer [12, 14, 16, 17]. We confirmed that the expression levels of LDHA were significantly higher in pancreatic cancer tissues (tumor) compared to their matched adjacent normal tissues (normal) (Figure 1C). Western blot analysis also showed that the expression levels of LDHA were significantly higher in colorectal cancer tissues (tumor) compared to their matched adjacent normal tissues (normal) (Figure 1D). Taken together, these results suggested that higher levels LDHA expressed in colorectal cancer tissues plays important roles in cancer progression.

### Knockdown of LDHA led to reduced cell growth *in vitro* and *in vivo*

**Figure 2:**
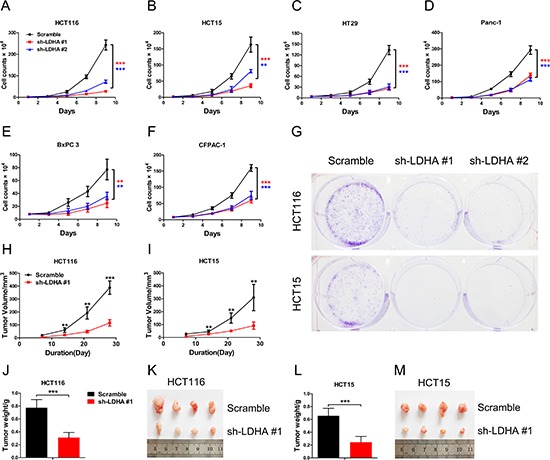
Knockdown of LDHA inhibits cell growth *in vitro* and *in vivo* **(A–F)** Cell proliferation assay results. Twenty thousand (A, B, D) or forty thousand (C, E, F) cells stably expressing LDHA-shRNA and scramble-shRNA were plated in 6-well plates and the cells were counted every alternate day and the numbers are plotted from three independent experimental wells. **(G)** Colony formation assay results. Five hundred HCT116 or HCT15 cells stably expressing LDHA-shRNA or scramble-shRNA were plated in 6-well plates supplemented with 10% FBS for 2 days followed with 2% FBS for a further 12 days. Colonies were stained with crystal violet. The number of colonies was counted from three independent experimental wells. **(H–M)** Tumor formation assay results. 6 × 10^6^ cells with indicated expression conditions were injected subcutaneously into 6 week-old nude mice (*n* = 8 mice/group). Tumors were monitored once a week. After four weeks, the mice were sacrificed, and the tumors were resected, weighed and imaged. Tumor volume, tumor weight, and tumor photos of HCT116 (H, J, K) or HCT15 (I, L, M) cells stably expressing LDHA-shRNA and scramble-shRNA were analyzed. Data represent mean ± SD. **0.001 < *p* < 0.01; ***p < 0.001.

To explore and confirm the role of LDHA in cancer cells, we deleted the expression of LDHA in colorectal cancer and pancreatic cancer cell lines by shRNA (Supplementary Figure S1). We found that knockdown of LDHA in CRC cell lines HCT116, HCT15, and HT29 can significantly inhibit cell growth (Figure 2A, 2B, and 2C). We also observed that there is a decreased cell proliferation in pancreatic cancer cells after deletion of LDHA (Figure 2D, 2E, and 2F). Furthermore, knockdown of LDHA in CRC cell lines HCT116 and HCT15 significantly reduced colony formation (Figure 2G). These results indicated that LDHA possesses protumorigenic role in CRC and pancreatic cancer cells.

To further investigate the biologic significance of LDHA in tumor growth, we performed xenograft experiments using the HCT116 and HCT15 stable cell lines with LDHA knockdown. Knockdown of LDHA in HCT116 and HCT15 cells led to a significant reduction in tumor size and tumor weight (Figure 2H–2M). Taken together, these data indicate that LDHA is essential for colorectal cancer cell growth *in vitro* and *in vivo*.

### Identification of differentially regulated miRNAs targeting LDHA

**Figure 3:**
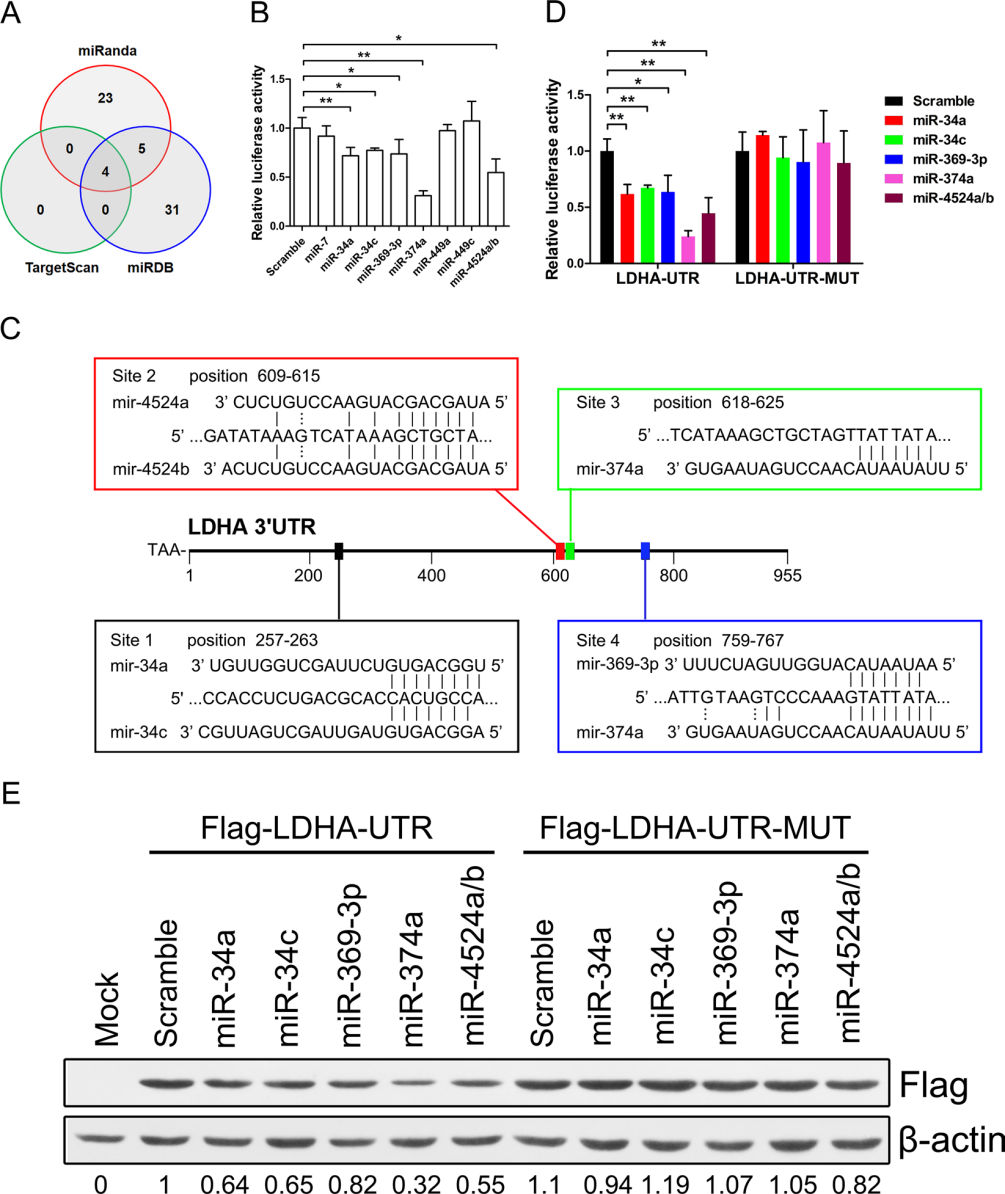
miR-34a, miR-34c, miR-369–3p, miR-374a, and miR-4524a/b suppress the expression of LDHA **(A)** Venn diagrams showing the number of potential miRNAs target 3′ UTR of LDHA as predicted by two databases: TargetScan and miRDB. **(B)** Dual-luciferase assay results. Repression of LDHA by candidate miRNAs was measured as ratios of Renilla and Firefly luciferase activity in 293T cells. The luciferase activity was quantified from five independent experimental wells. **(C)** Sequences of indicated miRNAs and their potential binding site at the 3′ UTR of LDHA. **(D)** Dual-luciferase assays showing repression of wild-type UTR (LDHA-UTR) or mutant UTR (LDHA-UTR-MUT) following transfection of indicated miRNAs or scramble. **(E)** Western blot analysis showing LDHA levels expressed from LDHA cDNA with a wild-type UTR (Flag-LDHA-UTR) or mutant UTR (Flag-LDHA-UTR-MUT) in the presence of indicated miRNAs or scramble in 293T cells. Data represent mean ± SD. *0.01 < *p* < 0.05; **0.001 < *p* < 0.01.

To identify miRNAs that directly bind to 3′ UTR of LDHA, we used mRNA target-predicting algorithms (TargetScan, miRanda, and miRDB). There are four miRNAs that overlapped among these three algorithms (Figure 3A). We selected these four miRNAs (miR-34a, miR-34c, miR-449a, and miR-449c) and other five miRNAs (miR-7, miR-369-3p, miR-374a, miR-4524a, and miR-4524b) that had one or more binding site in 3′ UTR of LDHA (Figure 3A, Supplementary Figure S2A).

To establish a direct relationship between these miRNAs and LDHA, we cloned the 3′ UTR of LDHA gene into a dual-luciferase UTR vector. Notably, 3′ UTR of LDHA appeared to be repressed by miR-miR34a, miR-34c, miR-369-3p, miR-374a, and miR-4524a/b (Figure 3B). Specifically, miR-374a suppressed the 3′ UTR of LDHA in a three-quarters level (Figure 3B). Evaluation of the 3′ UTR sequence of LDHA revealed one binding site for miR-34a, miR-34c, miR-369-3p, and miR-4524a/b, and two binding sites with perfect matches in the seed sequences for miR-374a (Figure 3C). We next generated mutations in the binding site to abrogate these miRNAs-LDHA 3′ UTR interaction (Supplementary Figure S2B). As expected, whereas a reporter with an integral LDHA 3′ UTR were effectively suppressed by miR-34a, miR-34c, miR-369-3p, miR-374a, and miR-4524a/b, that with LDHA 3′ UTR carrying mutated binding sites were refractory to suppression by these miRNAs (Figure 3D). miR-34a transactivated by p53 suppressed the LDHA expression in cancer cells (Supplementary Figure S2D). A number of key targets of miR-34a and miR-34c had been reported in the literature [27]. To determine if LDHA is a key target for miR-34a and miR-34c, we compared the expression level of 22 target genes of miR-34 in HCT116 and Panc-1 cells and found that the expression level of LDHA is much higher than other genes (Supplementary Figure S3A and S3B), which means LDHA is a key target gene of miR-34. To determine if LDHA with a 3′ mutant UTR (Flag-LDHA-UTR-MUT) was also refractory to these miRNAs-mediated suppression, we expressed a cDNA that harbored each mutation in the 3′ UTR containing these miRNAs binding sites and a flag label. This mutant UTR abolished these miRNAs-mediated suppression of the LDHA (Figure 3E). The miR-34 family have been proposed as critical modulators of the p53 pathway and potential tumor suppressors in human cancers [28–30]. To investigate if P53 induced the expression of miR-34a was sufficient to suppress LDHA, we treated Panc-1, HCT116, and HCT15 cells with doxorubicin (Dox). These Dox-activated P53 directly up-regulate miR-34a, but not miR-34c (Supplementary Figure S2C and S2D). Taken together, these results indicate that miR-34a, miR-34c, miR-369-3p, miR-374a and miR-4524a/b directly regulate LDHA expression through the binding site in the 3′ UTR.

### Identified rs18407893 at 11p15.4 in 3′-UTR of LDHA bound by miR-374a

**Figure 4:**
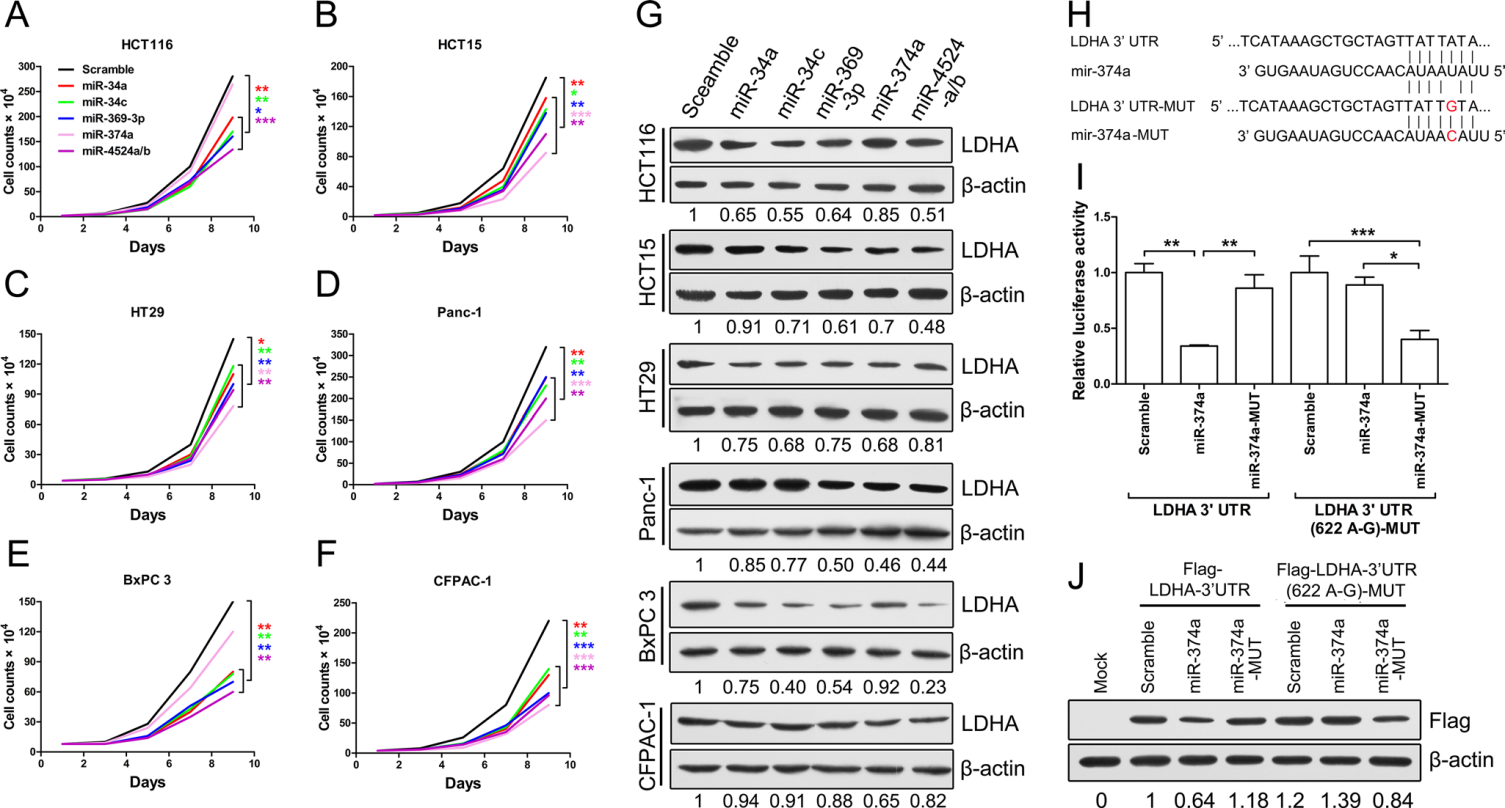
miR-34a, miR-34c, miR-369-3p, miR-374a, and miR-4524a/b inhibit cell growth through inhibition of LDHA **(A–F)** Cell proliferation assay results. Twenty thousand (A, B, D) or forty thousand (C, E, F) cells stably expressing indicated miRNAs or scramble cells were plated in 6-well plates and the cells were counted every alternate day and the numbers are plotted from three independent experimental wells. **(G)** Western blot analysis of LDHA in cells stably expressing indicated miRNAs or scramble cells. **(H)** Sequences of miR-374a and the binding site (position 618–625) at the 3′ UTR of LDHA. Red nucleotides shown in LDHA-3′ UTR-MUT was identified as rs18407893. **(I)** Dual-luciferase assays showing repression of wild-type UTR (LDHA-3′ UTR) or rs18407893 mutant UTR (LDHA-3′ UTR (622 A-G)) following transfection of scramble, miR-374a, or miR-374a-MUT. Data represent mean ± SD. **(J)** Western blot analysis showing LDHA levels expressed from LDHA cDNA with a wild-type UTR (Flag-LDHA-3′ UTR) or rs18407893 mutant UTR (Flag-LDHA-3′ UTR (622 A-G)) in the presence of miR-374a or scramble in 293T cells. *0.01 < *p* < 0.05; **0.001 < *p* < 0.01; ***p < 0.001.

To further explore roles of miR-34a, miR-34c, miR-369-3p, miR-374a and miR-4524a/b in cancer cells, we stably expressed these miRNAs using a lentiviral delivery system in human colorectal caner and pancreatic cancer cell lines (Supplementary Figure S4A–S4E), and found that most cell lines stably expressing these miRNAs exhibited decreased cell proliferation (Figure 4A–4F), except for miR-374a in HCT116 and BxPC 3 cells (Figure 4A, and 4E). The colony formation results also shown that ectopic expression of these miRNAs significantly reduced colony formation, except for miR-374a in HCT116 and BxPC 3 cells (Supplementary Figure S4F).

We then investigated the inhibitory effect of these miRNAs on LDHA in human colorectal caner and pancreatic cancer cell lines, and found that cells stably expressed miR-34a, miR-34c, miR-369-3p, miR-374a and miR-4524a/b decreased the protein level of LDHA in varying degrees (Figure 4G). Furthermore, we investigated the impact of combinations of these miRNAs on LDHA, and found that combinations of these miRNAs in HCT15 cells nearly remove the LDHA expression (Supplementary Figure S4G). Surprisingly, HCT116 and BxPC 3 cells stably expressed miR-374a have almost no effect on LDHA protein level (Figure 4G), which means that miR-374a can not target the 3′ UTR of LDHA in HCT116 and BxPC 3 cells. Thence, we supposed that the binding site for miR-374a in 3′ UTR of LDHA may generate mutations in HCT116 and BxPC 3 cells. To verify this hypothesis, we sequenced the LDHA 3′ UTR of HCT116, HCT15, HT29, Panc-1, BxPC 3, and CFPAC-1, and found that there present a point mutation in HCT116 and BxPC 3 cells (Supplementary Table 1). This genetic loci rs18407893 at 11p15.4 exactly located in the binding site for miR-374a in 3′ UTR of LDHA (Figure 4H). To better understand this point mutation (rs18407893), we next generated mutations in the binding site of LDHA (LDHA 3′ UTR-MUT) and miR-374a (miR-374a-MUT) to investigate the effect of rs18407893 on miR-374a-LDHA 3′ UTR interaction activity (Figure 4H). As expected, whereas a reporter with an intact LDHA 3′ UTR was effectively suppressed by miR-374a, that with LDHA 3′ UTR carrying a point mutated binding site (LDHA 3′ UTR-MUT) was refractory to suppression by miR-374a, but with LDHA 3′ UTR-MUT was effectively suppressed by miR-374a carrying a point mutation (miR-374a-MUT) (Figure 4I). we also expressed a LDHA cDNA that harbored this point mutation (rs18407893) in the 3′ UTR and a flag label and found This point mutant UTR abolished miR-374a-mediated suppression of the LDHA (Figure 4J). Taken together, these results demonstrate that the binding site of LDHA for miR-374a can generate point mutation, through which cancer cells escape from the inhibitory regulation of miR-374a.

### miR-34a, miR-34c, miR-369-3p, miR-374a, and miR-4524a/b suppress glycolysis through inhibition of LDHA

**Figure 5:**
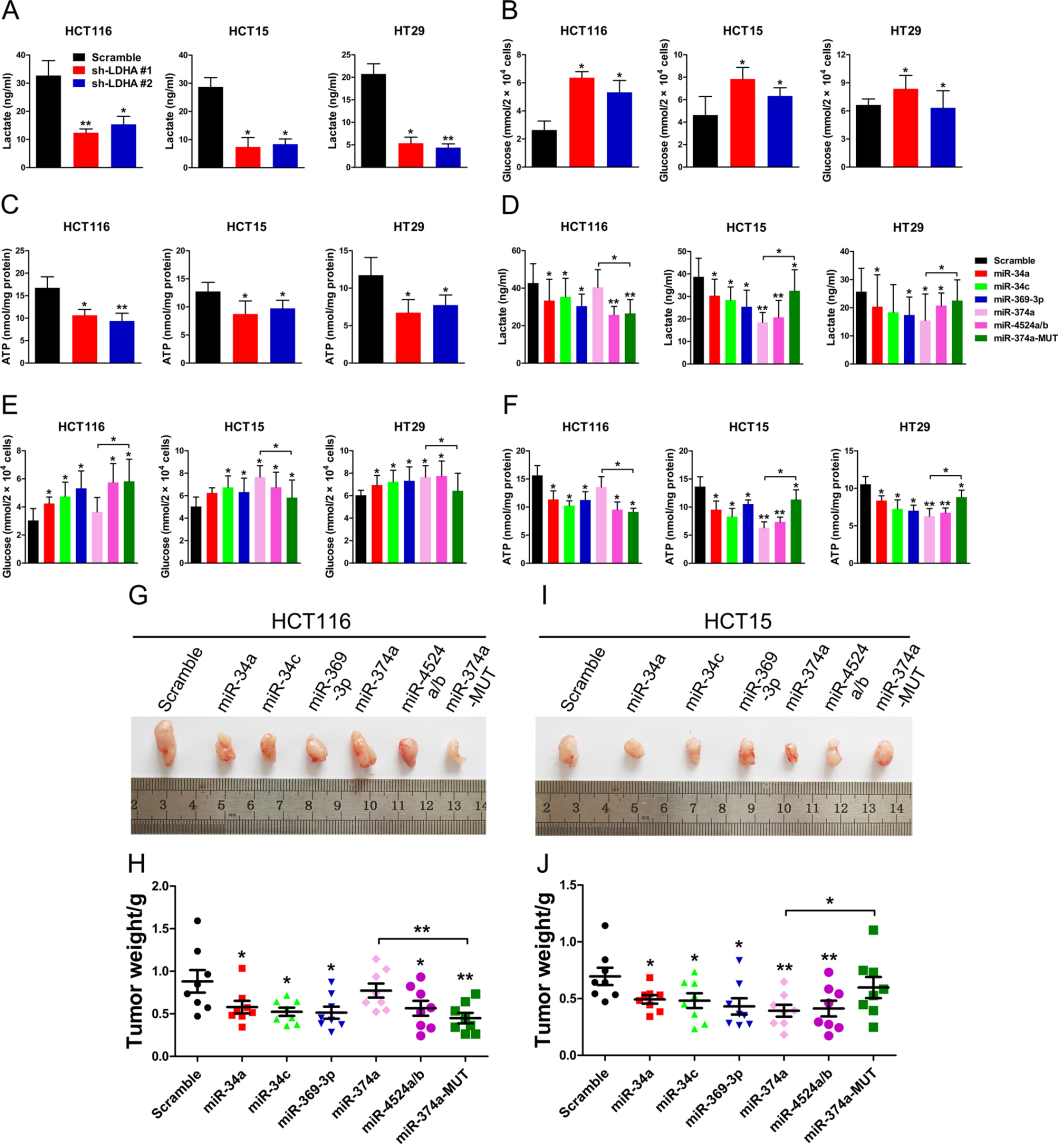
miR-34a, miR-34c, miR-369-3p, miR-374a, and miR-4524a/b suppress glycolysis and tumorigenesis **(A, B, C)** lactate (in the culture media) (A), Glucose (in the culture media) (B), and intracellular ATP levels (B) in human CRC cell lines stably expressing LDHA-shRNA and scramble-shRNA. **(D, E, F)** lactate (in the culture media) (D), Glucose (in the culture media) (E), and intracellular ATP levels (F) in human CRC cell lines stably expressing indicated miRNAs or scramble cells lines. **(G–J)** Tumor formation assay results. 6 × 10^6^ HCT116 or HCT15 cells with indicated expression conditions were injected subcutaneously into 6 week-old nude mice (*n* = 8 mice/group). After four weeks, the mice were sacrificed, and the tumors were resected, weighed and imaged. Tumor photos, and tumor weight of HCT116 (G, H) or HCT15 (I, J) cells stably expressing indicated miRNAs or scramble cells. Data represent mean ± SD. *0.01 < *p* < 0.05; **0.001 < *p* < 0.01.

To address the biologic significance of LDHA and relative miRNAs, especially rs18407893 located in the 3′ UTR of LDHA, we detected differences in metabolic parameters probably caused by LDHA knockdown. Depletion of LDHA levels in human CRC cells largely recapitulated the metabolic and bio-energetic patterns of CRC cells, e.g., decreased lactate production and glucose uptake, and decreased in intracellular ATP levels (Figure 5A, 5B, and 5C). Depletion of LDHA levels in human pancreatic cancer cells also influence aerobic glycolysis in, led to decreased lactate production and glucose uptake, and decreased in intracellular ATP levels (Supplementary Figure 5A, 5B, and 5C). In addition, High expression of miR-34a, miR-34c, miR-369-3p, miR-374a, and miR-4524a/b levels in human CRC and pancreatic cancer cell lines resulted in a consistent change of these metabolic parameters (Figure 5D, 5E, and 5F, Supplementary Figure 5D, 5E, and 5F). As expected, a modest increase in miR-374a levels in HCT116 and BxPC 3 cells failed to decrease lactate production, glucose uptake, and intracellular ATP levels (Figure 5D, 5E, and 5F, Supplementary Figure 5D, 5E, and 5F). However, enforced expression miR-374a-MUT in HCT116 and BxPC 3 cells resulted in a reversal of these metabolic parameters compared to miR-374a (Figure 5D, 5E, and 5F, Supplementary Figure 5D, 5E, and 5F). Furthermore, we found that combined expression of miR-34a, miR-34c, miR-369-3p, miR-374a, and miR-4524a/b in HCT116 or Panc-1 cells uncommonly led to decreased lactate production and glucose uptake, and decreased in intracellular ATP levels compared with expression of these miRNAs alone (Supplementary Figure 5G and 5H).

To evaluate the impact of miR-34a, miR-34c, miR-369-3p, miR-374a, miR-4524a/b, and rs18407893 on tumor growth, subcutaneous injections of HCT116 and HCT15 cells stably expressing miR-34a, miR-34c, miR-369-3p, miR-374a, miR-4524a/b, or miR-374a-MUT and scramble cells were performed in nude mice and the size of the resulting tumors were measured. There was a significant reduction in tumor size in these miRNAs overexpressed HCT116 (Figure 5G and 5H) and HCT15 cells (Figure 5I and 5J), except for miR-374a in HCT116 cells (Figure 5G and 5H). Inversely, HCT15 cells stably expressing miR-374a-MUT failed to suppress tumor growth (Figure 5I and 5J). Collectively, these data suggest that miR-34a, miR-34c, miR-369-3p, miR-374a, and miR-4524a/b suppresses glycolysis and tumor growth through inhibition of LDHA.

### Expression levels of miR-34a, miR-34c, miR-369-3p, miR-374a, and miR-4524a/b with LDHA in human CRC specimens

**Figure 6:**
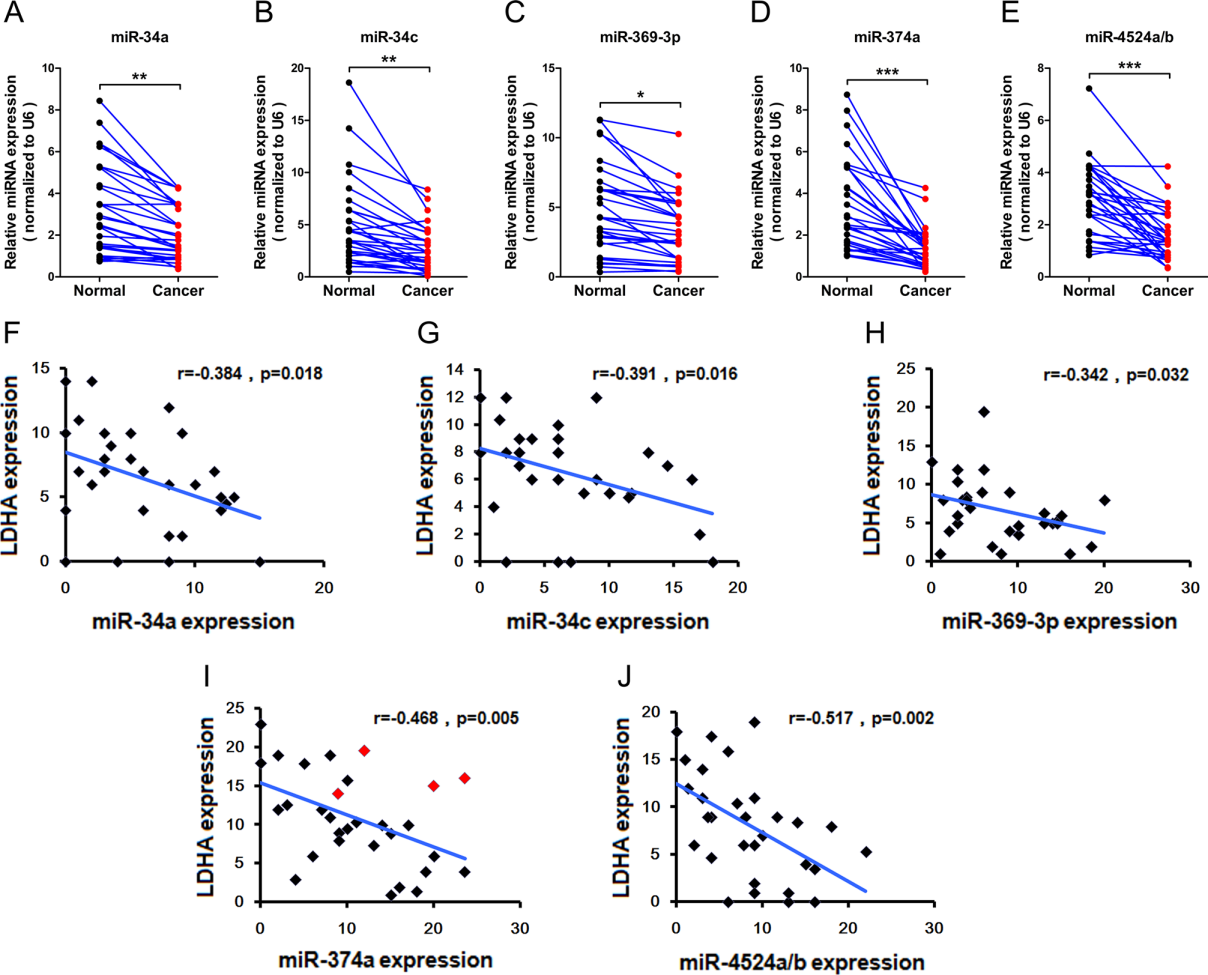
Expression levels of miR-34a, miR-34c, miR-369-3p, miR-374a, and miR-4524a/b with LDHA in human CRC tissues **(A–E)** miR-34a (A), miR-34c (B), miR-369–3p (C), miR-374a (D), and miR-4524a/b (E) were determined by RT-PCR in human colorectal cancer and adjacent normal tissues (*n* = 30). *0.01 < *p* < 0.05; **0.001 < *p* < 0.01; ***p < 0.001. **(F–J)** Correlative analysis of the indicated miRNAs, and LDHA in human colorectal cancer tissues (n = 30). Spearman correlation coefficient with the respective significance is indicated.

To test whether the regulations described above for CRC cell lines are also clinically relevant, we examined primary CRC specimens derived from 30 CRC patients and paired adjacent normal tissues. Indeed, we found that expression levels of miR-34a, miR-34c, and miR-369-3p in adjacent normal tissues were higher compared to primary CRC specimens (Figure 6A, 6B, and 6C). In addition, adjacent normal tissues has notable higher levels miR-374a, and miR-4524a/b than in primary CRC specimens (Figure 6D and 6E). Moreover, we analyzed the correlation between the level of LDHA and miRNAs in primary CRC specimens, and detected a negative correlation between LDHA levels and the expression of miR-34a (Figure 6F), miR-34c (Figure 6G), miR-369-3p (Figure 6H), miR-374a (Figure 6I), and miR-4524a/b (Figure 6J). Furthermore, we sequenced 3′ UTR of LDHA derived from 30 CRC patients, and found there are four cases (red icon) carrying the genetic loci, rs18407893 at 11p15.4 (in 3′-UTR of LDHA) bound by miR-374a (Figure 6I). The patients carrying rs18407893 had high levels of LDHA and miR-374a simultaneously (Figure 6I). Taken together, these results demonstrate that there is a negative correlation between LDHA levels and the expression of miR-34a, miR-34c, miR-369-3p, miR-374a, and miR-4524a/b in human CRC specimens.

## DISCUSSION

**Figure 7:**
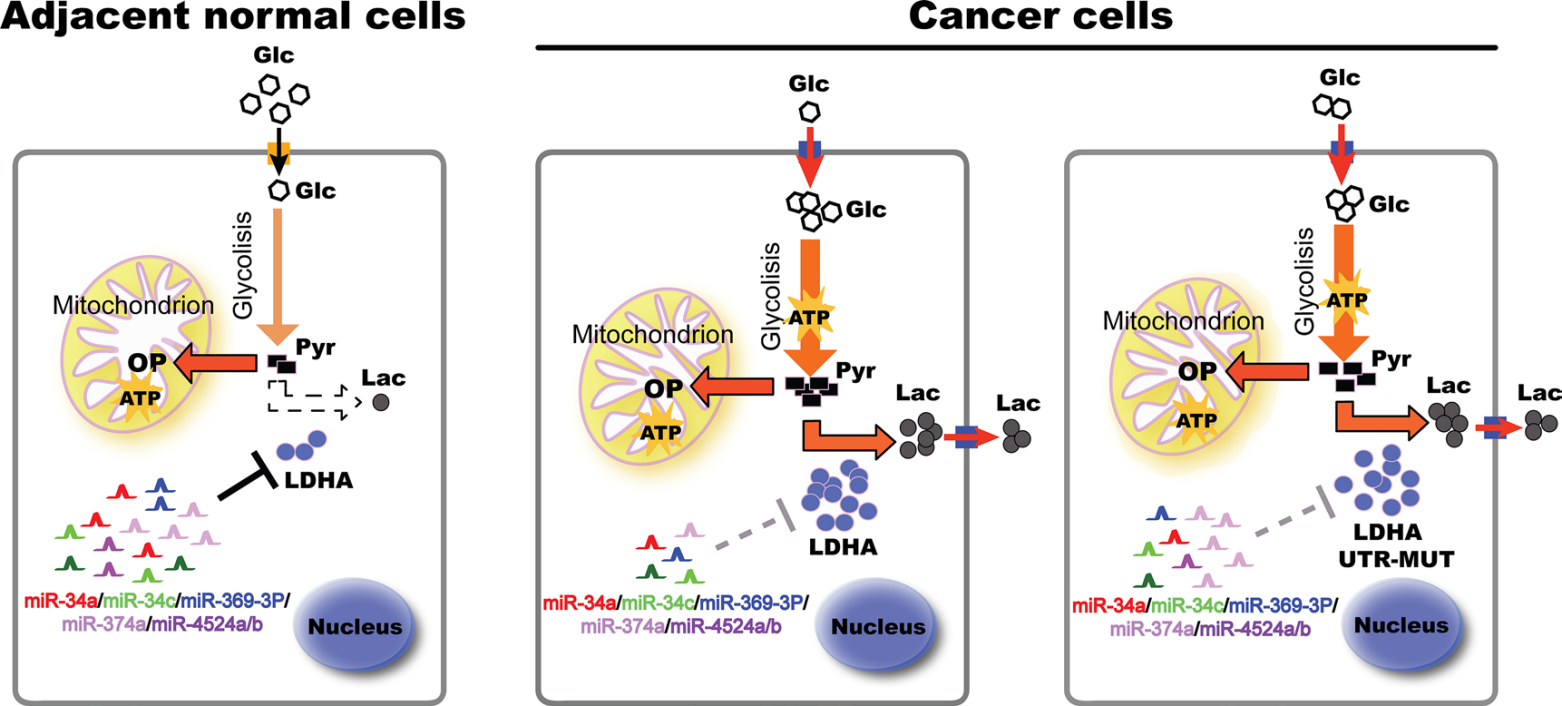
A model based on our studies In adjacent normal tissues, higher levels of miRNAs bind to LDHA mRNA 3′ UTR, and induce its degradation. In cancer tissues, decreased miRNAs lead to higher levels of LDHA, thereby accelerating glycolysis, lactate production, and ATP synthesis, promoting cell proliferation. Some cancer cells generate escape mechanism through mutation of miR-374a's binding site of LDHA, leading to warburg effect and cancer progression. Glc, glucose; Pyr, pyruvate; Lac, lactate; OP, oxidative phosphorylation; UTR, untranslated region.

Reprogramming of energy metabolism, including elevated glycolysis, is a near universal feature of cancer progression [31]. Cancer cells increases glucose uptake and metabolic intermediates for macromolecule biosynthesis to support rapid cell growth. Universally, glycolysis is highly elevated. Among the glycolytic enzymes, LDH is unique because it is necessary to maintain high glycolysis rate by producing NAD^+^ required in early steps in glycolysis [32, 33]. Advances in understanding the biology of tumor progression and metastasis have clearly highlighted the importance of aberrant tumor metabolism, which supports not only tumor cells' energy requirements but also their enormous biosynthetic needs. In this study, we uncovered the critical roles of LDHA in aerobic glycolysis in colorectal cancer cells and a mechanism of miRNAs regulated LDHA that contributes to its decreased protein level and activity (Figure 7). We demonstrate that LDHA knockdown significantly inhibits cell proliferation in human colorectal cancer cell lines both *in vitro* and *in vivo*. Similar results have been found using human pancreatic cancer cell lines. LDH converts pyruvate to lactate instead of generating acetyl-CoA for oxidative phosphorylation, a commonly observed phenomenon in many tumor cells. Indeed, LDHA knockdown leads to decreased lactate production and glucose uptake, and decreased in intracellular ATP levels in colorectal cancer cells.

LDHA upregulation is commonly observed in cancers, and reasons for LDHA upregulation in cancers is varied [9, 12, 14, 16, 22]. In pancreatic β cells, three microRNAs (miRNAs), miR-29a, miR-29b, and miR-124, selectively target both human and mouse LDHA 3′ untranslated regions. In addition, we found that miR-34a, miR-34c, miR-369-3p, miR-374a, and miR-4524a/b regulate LDHA levels by targeting LDHA 3′ untranslated regions (Figure 7). The miR-34 family was originally cloned and characterized in 2007 as a p53 target gene [30]. In recent years, miR-34 family, especially miR-34a, was identified as an important tumor suppressor [34–37]. p53 transactivated miR-34 suppresses the transcriptional activity of β-catenin-T-cell factor/lymphoid enhancer factor (TCF/LEF) complexes by targeting the untranslated regions of a set of highly-conserved targets in a network of Wnt pathway-regulated genes, including WNT1, WNT2, LRP6, β-catenin, and LEF1 [30, 34]. In colon cancer stem cells (CCSCs), miR34a demarcates self-renewal and differentiation by targeting Notch1 3′ untranslated regions [35]. Furthermore, miR-34a was under-expressed in CD44^+^ prostate cancer cells, and CD44 was identified and validated as a direct and functional target of miR-34a [36]. miR-34a as a novel and critical suppressor of osteoclastogenesis, bone resorption and the bone metastatic niche by directly targeting transforming growth factor-β-induced factor 2 (Tgif2) [37]. Kaller et al. combined pulsed SILAC (pSILAC) and microarray analyses and identified 1206 miR-34a-induced changes in protein and mRNA expression including LDHA, which implies miR-34a may involve in modulating glycolysis [25]. Accordingly, we systematically screen miRNAs to target LDHA 3′ untranslated regions.

It has been shown that alteration 3′ UTR of mRNA leads to an increase in protein translation, such as mutation and deletion [38–42]. A novel 3′ untranslated region (UTR) mutation was found to be associated with increased sensitivity to saracatinib and have a reduced affinity for miR-520a and miR-525a [38]. In gastric cancer, there is a frequent somatic mutation in CD274 3′ UTR leads to protein over-expression by disrupting miR-570 binding [39]. Yatsenko et al. show that in Drosophila the microRNA complex miR-310s acts as an executive mechanism to buffer levels of the muscular dystrophy-associated extracellular matrix receptor dystroglycan via its alternative 3′ UTR [41]. These studies indicated that alteration 3′ UTR of mRNA plays an important role in regulation of gene translation.

In this study, we identified a novel mutation (A–G transition) in HCT116 and BxPC 3 cell lines in the 3′ UTR that is located 622 base pairs from the stop codon of the LDHA gene. We also found this point mutation in human primary colorectal cancer tissues. These findings indicate that this mutation in the 3′ UTR of LDHA may alter the binding of miRNA(s), resulting in an increase in LDHA protein translation. Given this observation, we searched for potential miRNAs with a predicted sequence match for this region and identified miR-374a. Herein, we determined that miR-374a decreased luciferase reporter gene activity arising from wild-type 3′ UTR of LDHA but did not have an effect on the activity from the point mutant 3′ UTR sequence. These results show that we identified a genetic loci (located in the 3′ UTR sequence of LDHA) newly associated with increased colorectal cancer progression. Overall, we believe that our findings are hopefully translated into a biomarker-driven clinical trial.

At last, we show that the rs18407893 mutation can be suppressed by modified miR-374a, suggesting the possibility of restoring proper LDHA level, opening new strategies for the treatment of this type of mutations.

## MATERIALS AND METHODS

### Mice and animal studies

All animal experiments were conducted in accordance with a protocol approved by the Committee on the Use of Live Animals in Teaching and Research of the Tianjin Hospital. Female pathogen-free athymic nude mice (6-8-week-old) were purchased from the Academy of Military Medical Science (Beijing, China). HCT116, HCT15, HT29, Panc-1, Bxpc-3, and CFPAC-1 were purchased from American Type Culture Collection (ATCC). HCT116, HCT15, HT29, BxPC-3, and CFPAC-1 were cultured in 1640 (Hyclone) supplemented with 10% fetal bovine serum (FBS) (Hyclone) and 1% penicillin-streptomycin (Gibic). Panc-1 was cultured in DMEM (Hyclone) supplemented with 10% fetal bovine serum (FBS) (Hyclone) and 1% penicillin-streptomycin (Gibic). All cells were cultured at 37°C in an atmosphere of 5% CO2 in air.

### Detection of LDHA in human colon cancer tissues by tissue microarray (TMA)

Thirty surgically excised human CRC tissues and surrounding non-tumorous colonic tissues and 2 microarray slides containing paired colon cancer as well as non-cancerous tissues were obtained from the pathology Service of Logistics University of CAPF. The microarray slides were used to detect LDHA by immunohistochenistry. Briefly, the antigen was retrieved by high pressure and incubation in 0.01 M sodium citrate buffer. Then The slides were blocked in 10% normal goat serum in PBS and incubated at 4°C overnight in primary antibody solution of anti-LDHA (1:400). After being washed with 0.01 M PBS buffer, the slides were incubated with biotin-labeled species specific secondary antibodies and ABC complex for 30 min at 37°C. The slides were then developed with DAB (3, 3′-diaminobenzide tetrahydrochloride) substrate, counterstained with hematoxylin, and viewed under a microscope.

### Cloning of LDHA shRNA and pri-miRNA in lentiviral constructs, virus generation and transduction

To knockdown LDHA, several different shRNAs were used, human *LDHA* shRNA from Public TRC Portal were used: TRCN0000026537, TRCN0000162470, TRCN0000164922, TRCN0000166 246, TRCN0000166152 (#1), TRCN0000162267 (#2). The oligonucleotides were annealed and cloned into the AgeI/EcoR1 sites of the shRNA vector pLKO.1-puro (Addgene).

To express miR-34a, miR-34c, miR-369-3p, miR-374a, miR-449a/c, and miR-4524a/b in cells, about 500 bp of pri-miRNA containing the mature miRNAs sequence were respectively amplified and cloned into the PCDH-CMV-MCS-EF1-Puro lentiviral vector (Addgene). miRNAs were designed and cloned into the EcoR I/BamH I site of the lentiviral vector. Lentivirus was generated and titer was estimated in 293T cells by the serial dilution method. To select stable cell lines, viral supernatant was added to 10-cm dish for 24 hr, and then replaced with fresh medium for an additional 24 hr. Next, puromycin (2-10 μg/ml) was used to select stable cell lines.

### Cell proliferation assays

Briefly, cells were plated in 6-well plates at 2 × 10^4^ (HCT116, HCT15, and Panc-1) or 4 × 10^4^ (HT29 and BxPC-3) cells per well in triplicate. The cells were quantified and the numbers were plotted from three independent experimental wells for each condition and time point every alternate day.

### Colony formation assay

Briefly, one thousand cells were plated in 6-well plates supplemented with 10% FBS for 2 days followed with 2% FBS for a further 12 days. Colonies were stained with crystal violet and photographed.

### Measurement of glucose consumption, lactate production and ATP levels

To estimate the extracellular lactate concentration secreted by cancer cells, a Lactate Assay Kit (Cayman Chemical) was used according to the manufacturer's protocols. Then the lactate concentration of the supernatant was estimated using a standard lactate calibration curve prepared under the same condition and reported in a microplate reader.

### Animal experiments

1 × 10^7^ cells stably expressing scramble, LDHA sh-RNA, or pri-miRNA were injected subcutaneously into nude mice (8 mice per group). Tumor volumes were monitored one times a week for 4 weeks according to the following formula: TV = (Length) × (Width)^2^/2. Then, the tumor-bearing mice were killed, and their tumors were removed, weighed, and photographed.

### RNA isolation and quantitative RT-PCR analysis

Total RNA was isolated using RNeasy Plus Mini Kit (Qiagen) according to the manufacturer's protocols. Total RNA (2 μg) was used for synthesis of first-strand cDNA using Maxima First Strand cDNA Synthesis Kits for RT-qPCR (Thermo). Quantitative real-time PCR was performed using the SYBR green mix (Roche). The reactions were performed with a 7500 fast.

### Western blotting analysis

Antibody for LDHA was purchased from Cell Signaling. β-actin was purchased from Sungene (Tianjin, China). Whole cell lysate was prepared using RIPA lysis buffer in the presence of protease inhibitors. Total cell lysates were separated using 8%–12% SDS-PAGE, transferred onto PVDF membranes (Roche), and then detected using various primary antibodies. The antibody-antigen complexes were detected using the Chemiluminescent HRP Substrate (Millipore).

### 3′-UTR luciferase assay

The 3′-UTR of LDHA was amplified by PCR using cDNA from 293T cells and cloned into a p-mirGLO Dual-Luciferase miRNA Target Expression Vector (Promega). The miRNAs precursor expression vector and pmirGLO Dual-Luciferase 3′-UTR vector were co-transfected into 293T cells using X-tremeGENE HP DNA Transfection Reagent (Roche). Cells were harvested and lysed at 48 hr post-transfection. The interaction between miRNAs and 3′-UTR of LDHA was measured by Dual-luciferase assay system (Promega).

### Statistical analysis

All results were derived from at least three independent experiments. The data were analyzed using Microsoft Office Excel 2007 or IBM SPSS Statistics 19 and expressed as the mean ± SD using the GraphPad Prism statistical program. Differences with *p* < 0.05 were statistically significant.

## SUPPLEMENTARY FIGURES AND TABLES



## References

[1] Siegel R, Naishadham D, Jemal A (2013). Cancer statistics, 2013. CA: a cancer journal for clinicians.

[2] Jemal A, Bray F, Center MM, Ferlay J, Ward E, Forman D (2011). Global cancer statistics. CA: a cancer journal for clinicians.

[3] Davies JM, Goldberg RM (2011). Treatment of metastatic colorectal cancer. Seminars in oncology: Elsevier.

[4] Gatenby RA, Gillies RJ (2004). Why do cancers have high aerobic glycolysis?. Nature Reviews Cancer.

[5] Kim JW, Dang CV (2006). Cancer's molecular sweet tooth and the Warburg effect. Cancer research.

[6] Dang CV, Le A, Gao P (2009). MYC-induced cancer cell energy metabolism and therapeutic opportunities. Clinical cancer research : an official journal of the American Association for Cancer Research.

[7] Kaelin WG, Ratcliffe PJ (2008). Oxygen sensing by metazoans: the central role of the HIF hydroxylase pathway. Mol Cell.

[8] Tennant DA, Durán RV, Gottlieb E (2010). Targeting metabolic transformation for cancer therapy. Nature Reviews Cancer.

[9] Kambe T, Tada J, Chikuma M, Masuda S, Nagao M, Tsuchiya T, Ratcliffe PJ, Sasaki R (1998). Embryonal carcinoma P19 cells produce erythropoietin constitutively but express lactate dehydrogenase in an oxygen-dependent manner. Blood.

[10] Luo W, Semenza GL (2011). Pyruvate kinase M regulates glucose metabolism by functioning as a coactivator for hypoxia-inducible factor 1 in cancer cells. Oncotarget.

[11] Chung F-Z, Tsujibo H, Bhattacharyya U, Sharief FS, Li S (1985). Genomic organization of human lactate dehydrogenase-A gene. Biochem J.

[12] Shi M, Cui J, Du J, Wei D, Jia Z, Zhang J, Zhu Z, Gao Y, Xie K (2014). A novel KLF4/LDHA signaling pathway regulates aerobic glycolysis in and progression of pancreatic cancer. Clin Cancer Res.

[13] Mirebeau-Prunier D, Le Pennec S, Jacques C, Fontaine JF, Gueguen N, Boutet-Bouzamondo N, Donnart A, Malthiery Y, Savagner F (2013). Estrogen-related receptor alpha modulates lactate dehydrogenase activity in thyroid tumors. PloS one.

[14] Zhao D, Zou SW, Liu Y, Zhou X, Mo Y, Wang P, Xu YH, Dong B, Xiong Y, Lei QY, Guan KL (2013). Lysine-5 acetylation negatively regulates lactate dehydrogenase A and is decreased in pancreatic cancer. Cancer Cell.

[15] Yang Y, Su D, Zhao L, Zhang D, Xu J, Wan J, Fan S, Chen M (2014). Different effects of LDH-A inhibition by oxamate in non-small cell lung cancer cells. Oncotarget.

[16] Cui J, Shi M, Xie D, Wei D, Jia Z, Zheng S, Gao Y, Huang S, Xie K (2014). FOXM1 promotes the warburg effect and pancreatic cancer progression via transactivation of LDHA expression. Clin Cancer Res.

[17] Rong Y, Wu W, Ni X, Kuang T, Jin D, Wang D, Lou W (2013). Lactate dehydrogenase A is overexpressed in pancreatic cancer and promotes the growth of pancreatic cancer cells. Tumour biology : the journal of the International Society for Oncodevelopmental Biology and Medicine.

[18] Girgis H, Masui O, White NM, Scorilas A, Rotondo F, Seivwright A, Gabril M, Filter ER, Girgis AH, Bjarnason GA (2014). Lactate Dehydrogenase A is a potential prognostic marker in clear cell renal cell carcinoma. Molecular Cancer.

[19] Yao F, Zhao T, Zhong C, Zhu J, Zhao H (2013). LDHA is necessary for the tumorigenicity of esophageal squamous cell carcinoma. Tumour biology : the journal of the International Society for Oncodevelopmental Biology and Medicine.

[20] Cai Z, Zhao J-S, Li J-J, Peng D-N, Wang X-Y, Chen T-L, Qiu Y-P, Chen P-P, Li W-J, Xu L-Y (2010). A combined proteomics and metabolomics profiling of gastric cardia cancer reveals characteristic dysregulations in glucose metabolism. Molecular & Cellular Proteomics.

[21] White N, Masui O, DeSouza LV, Krakovska O, Metias S, Romaschin AD, Honey RJ, Stewart R, Pace K, Lee J (2014). Quantitative proteomic analysis reveals potential diagnostic markers and pathways involved in pathogenesis of renal cell carcinoma. Oncotarget.

[22] Qing G, Skuli N, Mayes PA, Pawel B, Martinez D, Maris JM, Simon MC (2010). Combinatorial regulation of neuroblastoma tumor progression by N-Myc and hypoxia inducible factor HIF-1alpha. Cancer Res.

[23] Shim H, Dolde C, Lewis BC, Wu C-S, Dang G, Jungmann RA, Dalla-Favera R, Dang CV (1997). c-Myc transactivation of LDH-A: implications for tumor metabolism and growth. Proceedings of the National Academy of Sciences.

[24] Zhao D, Xiong Y (2013). LDH-A acetylation: implication in cancer. Oncotarget.

[25] Kaller M, Liffers S-T, Oeljeklaus S, Kuhlmann K, Röh S, Hoffmann R, Warscheid B, Hermeking H (2011). Genome-wide characterization of miR-34a induced changes in protein and mRNA expression by a combined pulsed SILAC and microarray analysis. Molecular & Cellular Proteomics.

[26] Pullen TJ, da Silva Xavier G, Kelsey G, Rutter GA (2011). miR-29a and miR-29b contribute to pancreatic beta-cell-specific silencing of monocarboxylate transporter 1 (Mct1). Molecular and cellular biology.

[27] Hermeking H (2010). The miR-34 family in cancer and apoptosis. Cell Death Differ.

[28] Concepcion CP, Han YC, Mu P, Bonetti C, Yao E, D'Andrea A, Vidigal JA, Maughan WP, Ogrodowski P, Ventura A (2012). Intact p53-dependent responses in miR-34- deficient mice. PLoS genetics.

[29] Siemens H, Jackstadt R, Kaller M, Hermeking H (2013). Repression of c-Kit by p53 is mediated by miR-3 and is associated with reduced chemoresistance, migration and stemness. Oncotarget.

[30] Chang TC, Wentzel EA, Kent OA, Ramachandran K, Mullendore M, Lee KH, Feldmann G, Yamakuchi M, Ferlito M, Lowenstein CJ, Arking DE, Beer MA, Maitra A, Mendell JT (2007). Transactivation of miR-34a by p53 broadly influences gene expression and promotes apoptosis. Mol Cell.

[31] Hanahan D, Weinberg RA (2011). Hallmarks of cancer: the next generation. Cell.

[32] Bui T, Thompson CB (2006). Cancer's sweet tooth. Cancer cell.

[33] Vander Heiden MG, Cantley LC, Thompson CB (2009). Understanding the Warburg effect: the metabolic requirements of cell proliferation. science.

[34] Kim NH, Kim HS, Kim NG, Lee I, Choi HS, Li XY, Kang SE, Cha SY, Ryu JK, Na JM, Park C, Kim K, Lee S, Gumbiner BM, Yook JI, Weiss SJ (2011). p53 and microRNA-3 are suppressors of canonical Wnt signaling. Sci Signal.

[35] Bu P, Chen KY, Chen JH, Wang L, Walters J, Shin YJ, Goerger JP, Sun J, Witherspoon M, Rakhilin N, Li J, Yang H, Milsom J, Lee S, Zipfel W, Jin MM (2013). A microRNA miR-34a-regulated bimodal switch targets Notch in colon cancer stem cells. Cell Stem Cell.

[36] Liu C, Kelnar K, Liu B, Chen X, Calhoun-Davis T, Li H, Patrawala L, Yan H, Jeter C, Honorio S, Wiggins JF, Bader AG, Fagin R, Brown D, Tang DG (2011). The microRNA miR-34a inhibits prostate cancer stem cells and metastasis by directly repressing CD44. Nat Med.

[37] Krzeszinski JY, Wei W, Huynh H, Jin Z, Wang X, Chang TC, Xie XJ, He L, Mangala LS, Lopez-Berestein G, Sood AK, Mendell JT, Wan Y (2014). miR-34a blocks osteoporosis and bone metastasis by inhibiting osteoclastogenesis and Tgif2. Nature.

[38] Arcaroli JJ, Quackenbush KS, Powell RW, Pitts TM, Spreafico A, Varella-Garcia M, Bemis L, Tan AC, Reinemann JM, Touban BM, Dasari A, Eckhardt SG, Messersmith WA (2012). Common PIK3CA Mutants and a Novel 3′ UTR Mutation Are Associated with Increased Sensitivity to Saracatinib. Clinical Cancer Research.

[39] Wang W, Sun J, Li F, Li R, Gu Y, Liu C, Yang P, Zhu M, Chen L, Tian W, Zhou H, Mao Y, Zhang L, Jiang J, Wu C, Hua D (2012). A frequent somatic mutation in CD274 3′-UTR leads to protein over-expression in gastric cancer by disrupting miR-570 binding. Hum Mutat.

[40] Simon D, Laloo B, Barillot M, Barnetche T, Blanchard C, Rooryck C, Marche M, Burgelin I, Coupry I, Chassaing N, Gilbert-Dussardier B, Lacombe D, Grosset C, Arveiler B (2010). A mutation in the 3′-UTR of the HDAC6 gene abolishing the post-transcriptional regulation mediated by hsa-miR-433 is linked to a new form of dominant X-linked chondrodysplasia. Hum Mol Genet.

[41] Yatsenko AS, Marrone AK, Shcherbata HR (2014). miRNA-based buffering of the cobblestone-lissencephaly-associated extracellular matrix receptor dystroglycan via its alternative 3′-UTR. Nat Commun.

[42] Dini Modigliani S, Morlando M, Errichelli L, Sabatelli M, Bozzoni I (2014). An ALS-associated mutation in the FUS 3′-UTR disrupts a microRNA-FUS regulatory circuitry. Nat Commun.

