# Supplementary material for: Lactate dehydrogenase A negatively regulated by miRNAs promotes aerobic glycolysis and is increased in colorectal cancer
Source: Oncotarget. 2015 Apr 10;6(23):19456–68. doi: 10.18632/oncotarget.3318 (PMC4637298; doi:10.18632/oncotarget.3318)
Supplement: Supplementary file 1 [file oncotarget-06-19456-s001.pdf]

SUPPLEMENTARY FIGURES AND TABLES

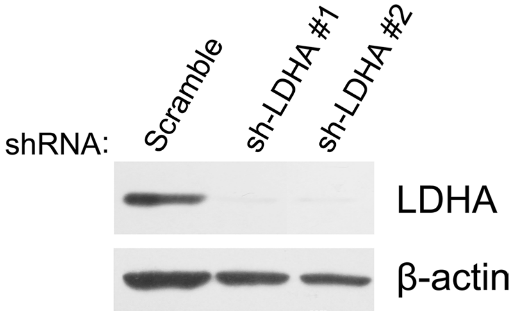

Supplementary Figure 1: Western blot analysis of LDHA in cells stably expressing LDHA-shRNA or scramble-shRNA.

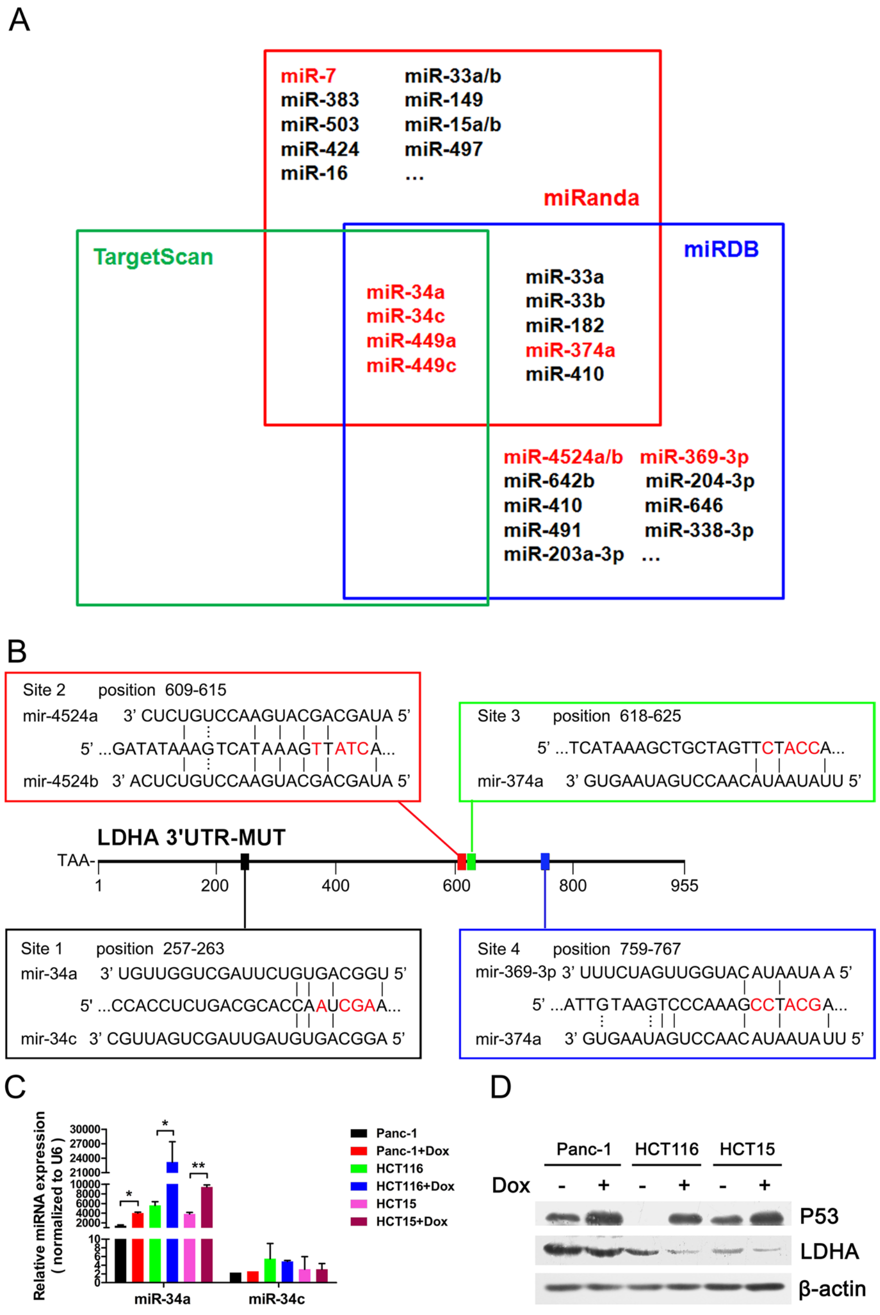

Supplementary Figure 2: (A) miRNAs, predicted by miRanda, TargetScan and miRDB, target LDHA. miRNAs that were predicted by two or more algorithms are indicated. **(B)** Sequences of indicated miRNAs and nucleotides mutated in LDHA-3′ UTR-MUT. **(C)** RT-PCR analysis of miR-34a and miR-34c in Panc 1, HCT116, and HCT15 cells treated with doxorubicin. **(D)** Western blot analysis of P53 and LDHA in Panc 1, HCT116, and HCT15 cells treated with doxorubicin.

A

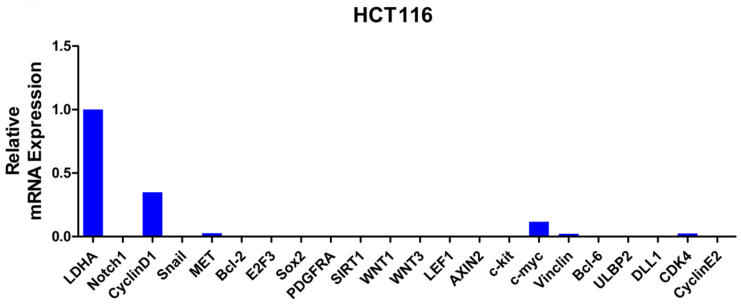

B

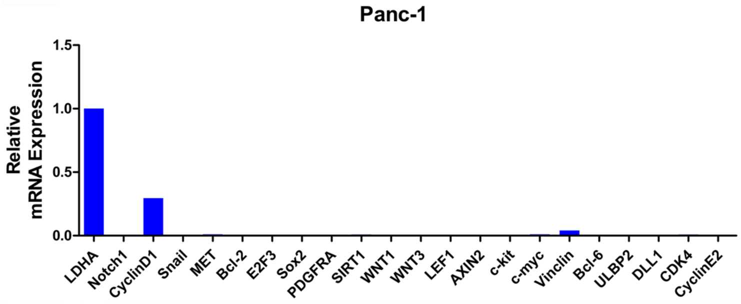

Supplementary Figure 3: (A, B) RT-PCR analysis of the target genes of miR-34 in HCT116 (A) or Panc-1 (B) cells.

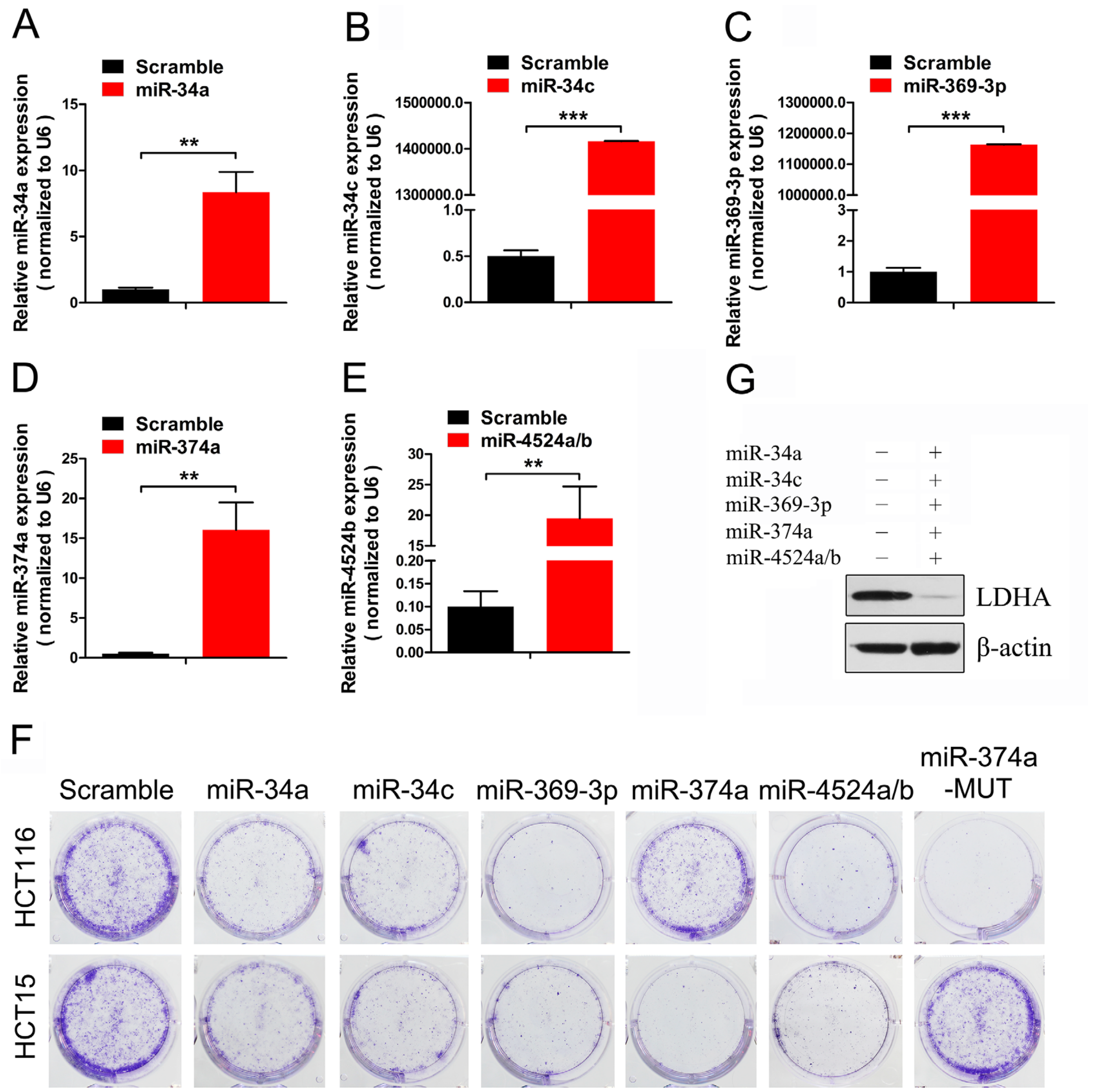

Supplementary Figure 4: (A–E) RT-PCR analysis of miR-34a **(A),** miR-34c **(B),** miR-369–3p **(C),** miR-374a **(D),** and miR-4524a/b **(E)** in cells stably expressing indicated miRNA. **(F)** Colony formation assay results. Five hundred HCT116 or HCT15 cells stably expressing indicated miRNAs or scramble cells were plated in 6-well plates supplemented with 10% FBS for 2 days followed with 2% FBS for a further 12 days. Colonies were stained with crystal violet. The number of colonies was counted from three independent experimental wells. **(G)** Western blot analysis of LDHA in HCT15 cells stably expressing indicated miRNAs.

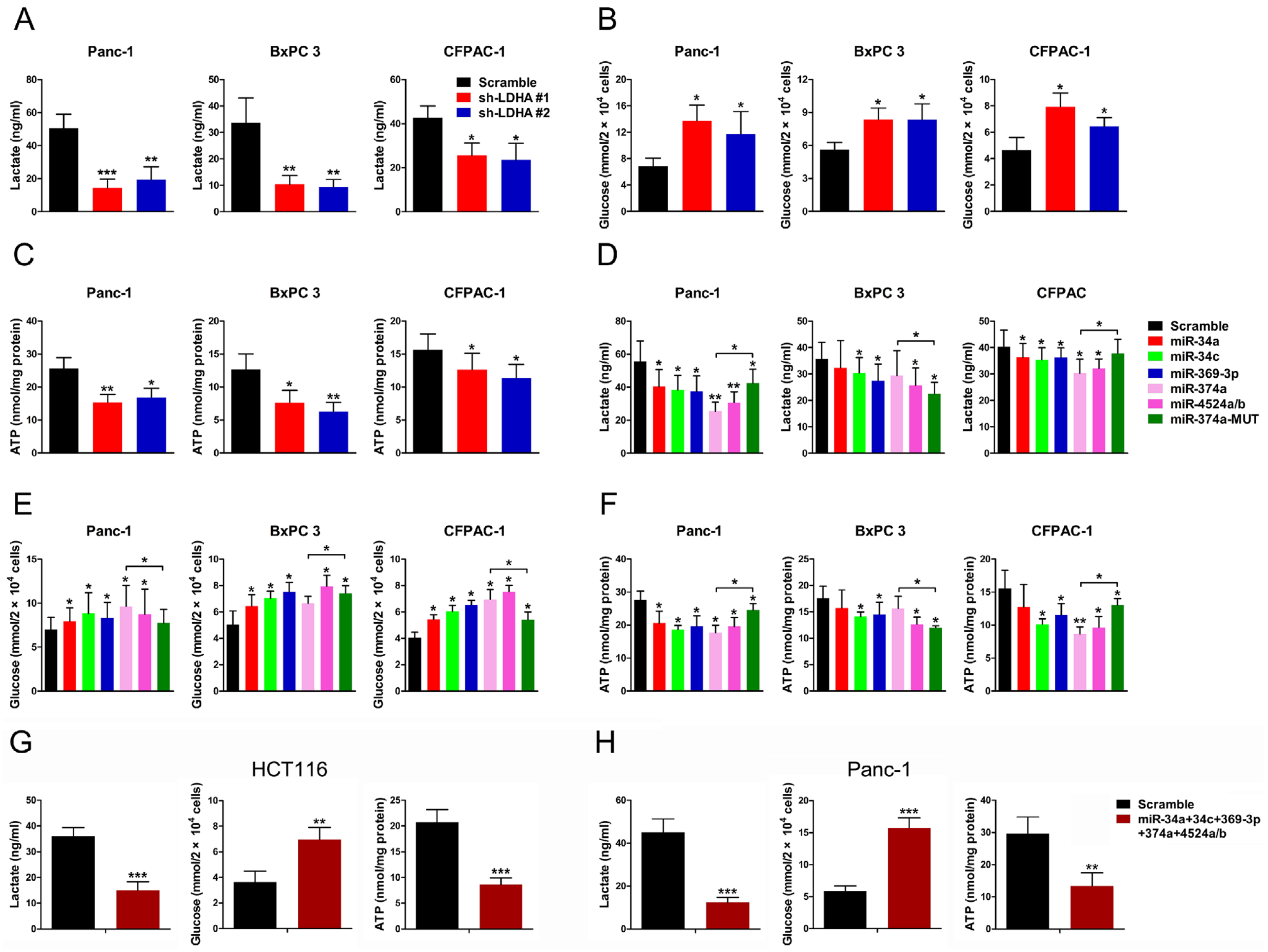

Supplementary Figure 5: (A, B, C) Lactate (in the culture media) **(A),** Glucose (in the culture media) **(B),** and intracellular ATP levels **(C)** in human pancreatic cancer cell lines stably expressing LDHA-shRNA and scramble-shRNA. **(D, E, F)** Lactate (in the culture media) **(D),** Glucose (in the culture media) **(E),** and intracellular ATP levels **(F)** in human pancreatic cancer cell lines stably expressing indicated miRNAs or scramble cells lines. **(G, H)** Analysis of Lactate (in the culture media), Glucose (in the culture media), and intracellular ATP levels in HCT116 **(G)** or Panc-1 **(H) c**ells stably expressing miR-34a, miR-34c, miR-369–3p, miR-374a, and miR-4524a/b compared with scramble cells.

Supplementary Table 1: The sequence in position 618–625 of LDHA 3′ UTR

Cell lines	Sequence
HCT116	TATTGAT
HCT15	TATTATA
HT29	TATTATA
Panc-1	TATTATA
BxPC 3	TATTGAT
CFPAC-1	TATTATA

UTR: untranslated region

Supplementary Table 2: List of primer

Name of primer	Sequences of primer
RT-LDHA-F	TTGACCTACGTGGCTTGGAAG
RT-LDHA-R	GGTAACGGAATCGGGCTGAAT
3′-UTR-LDHA-F	GCGTTTAAACCCTGTCTAGGCTACAACAGGAT
3′-UTR-LDHA-R	CCCTCGAGTTGTTACCAGCTTCCAGAGGA
Pri-miR-34a-F	GCTCTAGATTCCGCACCCACGAGCAG
Pri-miR-34a-R	CGGAATTCCCTTCTTATCAACAGGTGCTGG
Pri-miR-34c-F	GCTCTAGATCACTGTGTCTATTTGCCATCGTC
Pri-miR-34c-R	ATTTGCGGCCGCATAGGCAATGGATGAAAT
Pri-miR-369–3p-F	GCGAATTCTTCCACCAGGGAGGCCGTC
Pri-miR-369–3p-R	ATTTGCGGCCGCCAAGGTGAGATTTGATACT
Pri-miR-374a-F	GGTCTAGAAGCTGACAATCCAGAGCCTGT
Pri-miR-374a-R	CGGAATTCGGGTTAGGGAAAGCCTGAATAT
Pri-miR-449a/c-F	GCTCTAGAGCACCATTATAAAACTGTGCATTG
Pri-miR-449a/c-R	GCGAATTCGTTTTTGACTAGATTCAAGCAAAT
Pri-miR-4524a/b-F	GCTCTAGAGCAACACAATGGCATTAGGAAGC
Pri-miR-4524a/b-R	ATTTGCGGCCGCTTTATAAACAGGTCTGCTGCAC
Flag-LDHA-F	CCGGAATTCGCCACCATGGATTACAAGGATGACG ACGATAAGGCAACTCTAAAGGATCAGCT
Flag-LDHA-R	CGCGGATCCTTGTTACCAGCTTCCAGAGGAC
miR-34a stem loop	GTCGTATCCAGTGCAGGGTCCGAGGTATTCGC ACTGGATACGACACAACC
RT- miR-34a-F	GGTCCTGGCAGTGTCTTA
miR-34c stem loop	GTCGTATCCAGTGCAGGGTCCGAGGTATTCGC ACTGGATACGACGCAATC
RT- miR-34c-F	GGTCCAGGCAGTGTAGTTAGCT
miR-369–3p stem loop	GTCGTATCCAGTGCAGGGTCCGAGGTATTCGCAC TGGATACGACAAAGAT
RT- miR-369–3p-F	GGTCCAATAATACATGGTTG
miR-374a stem loop	GTCGTATCCAGTGCAGGGTCCGAGGTATTCGCAC TGGATACGACCACTTA
RT- miR-374a-F	GGTCCTTATAATACAACCTGA
miR-4524a stem loop	GTCGTATCCAGTGCAGGGTCCGAGGTATTCGCAC TGGATACGACTGAGAC
RT- miR-4524a-F	GGTCCATAGCAGCATGAACCT
miR-4524b stem loop	GTCGTATCCAGTGCAGGGTCCGAGGTATTCG CACTGGATACGACGAGACA
RT- miR-4524b-F	GGTCCATAGCAGCATAAGCC

(*Continued*)

Name of primer	Sequences of primer
RT-miRNA-R	GTGCAGGGTCCGAGGT
RT-Notch1-F	TGGACCAGATTGGGGAGTTC
RT-Notch1-R	GCACACTCGTCTGTGTTGAC
RT-CyclinD1-F	CAATGACCCCGCACGATTTC
RT-CyclinD1-R	CATGGAGGGCGGATTGGAA
RT-Snail-F	ACTGCAACAAGGAATACCTCAG
RT-Snail-R	GCACTGGTACTTCTTGACATCTG
RT-Met-F	GGTTCACTGCATATTCTCCCC
RT-Met-R	ACCATCTTTCGTTTCCTTTAGCC
RT-Bcl 2-F	GGTGGGGTCATGTGTGTGG
RT-Bcl 2-R	CGGTTCAGGTACTCAGTCATCC
RT-E2F3-F	GTATGATACGTCTCTTGGTCTGC
RT-E2F3-R	CAAATCCAATACCCCATCGGG
RT-Sox2-F	TACAGCATGTCCTACTCGCAG
RT-Sox2-R	GAGGAAGAGGTAACCACAGGG
RT-PDGFRA-F	TTGAAGGCAGGCACATTTACA
RT-PDGFRA-R	GCGACAAGGTATAATGGCAGAAT
RT-SIRT1-F	AGCCAATTCTTTTTGTGTTCGTG
RT-SIRT1-R	CGGCGTTTATCTTCGCTATCA
RT-WNT1-F	CGGCGTTTATCTTCGCTATCA
RT-WNT1-R	GCAGGATTCGATGGAACCTTCT
RT-WNT3-F	AGGGCACCTCCACCATTTG
RT-WNT3-R	GACACTAACACGCCGAAGTCA
RT-LEF1-F	TGCCAAATATGAATAACGACCCA
RT-LEF1-R	GAGAAAAGTGCTCGTCACTGT
RT-AXIN2-F	TACACTCCTTATTGGGCGATCA
RT-AXIN2-R	TTGGCTACTCGTAAAGTTTTGGT
RT-c-Kit-F	ACTTGAGGTTTATTCCTGACCCC
RT-c-Kit-R	GCAGACAGAGCCGATGGTAG
RT-c-myc-F	GTCAAGAGGCGAACACACAAC
RT-c-myc-R	TTGGACGGACAGGATGTATGC
RT-Vinculin-F	ATGGGTCAAGGGGCATCCT
RT-Vinculin-R	GGCCCAAGATTCTTTGTGTAAGT
RT-Bcl 6-F	ACACATCTCGGCTCAATTTGC
RT-Bcl 6-R	AGTGTCCACAACATGCTCCAT
RT-ULBP2-F	GTGGTGGACATACTTACAGAGC

(*Continued*)

Name of primer	Sequences of primer
RT-ULBP2-R	CTGCCCATCGAAACTGAACTG
RT-DLL1-F	TGTGACGAGTGTATCCGCTAT
RT-DLL1-R	GTGTGCAGTAGTTCAGGTCCT
RT-CDK4-F	CTGGTGTTTGAGCATGTAGACC
RT-CDK4-R	GATCCTTGATCGTTTCGGCTG
RT- CyclinE2-F	TCAAGACGAAGTAGCCGTTTAC
RT- CyclinE2-R	TGACATCCTGGGTAGTTTTCCTC
